# The Effect of Branched Chain Amino Acid Supplementation on Stroke-Related Sarcopenia

**DOI:** 10.3389/fneur.2022.744945

**Published:** 2022-03-11

**Authors:** Min Kyu Park, Sook Joung Lee, Eunseok Choi, Sangjee Lee, JungSoo Lee

**Affiliations:** ^1^Department of Clinical Pharmacology and Therapeutics, College of Medicine, Chungbuk National University Hospital, Cheongju-si, South Korea; ^2^Department of Physical Medicine and Rehabilitation, Daejeon St. Mary's Hospital, College of Medicine, The Catholic University of Korea, Seoul, South Korea

**Keywords:** stroke, sarcopenia, rehabilitation, branched-chain amino acids, skeletal muscle index, function

## Abstract

**Background:**

Stroke-related sarcopenia is caused by various factors, such as brain damage, systemic catabolic state, skeletal muscle imbalance, and malnutrition. In the long-term care plan after stroke, appropriate rehabilitation strategies to achieve maximum functional improvement and prevent the development of sarcopenia are important. This study has investigated the effect of branched-chain amino acid (BCAA) supplementation on sarcopenia after stroke. We also evaluated the effect of BCAA on functional improvement during the intensive rehabilitation period.

**Methods:**

Patients with subacute stroke with stroke-related disabilities were enrolled and given dietary supplement powder containing BCAAs for 1 month. These BCAAs were supplied through the nutrition team during feeding time. Patients whose age, sex, and stroke lesions were similar to those of the study group were enrolled in the control group through medical record review. Both groups received personalized intensive inpatient rehabilitation therapy in a single-unit rehabilitation center. All patients' target calories were calculated regularly by the nutritional support team in our institution. Sarcopenia status was evaluated using grip strength and the skeletal muscle index (SMI), which was assessed by dual-energy X-ray absorptiometry (DEXA). The functional status associated with stroke was evaluated every month, including activities of daily living, balance, gait, and swallowing.

**Results:**

A total of 54 patients were enrolled, with 27 patients in each of the two groups. The study group showed significantly greater improvement in SMI after intervention than the control group. Both groups improved functionally over time, but the improvement in the study group was significantly greater than that in the control group. Univariate analysis revealed that patients with better functional status had a greater SMI with a combination of BCAA supplementation and intensive rehabilitation therapy.

**Conclusion:**

Our results showed a positive effect of BCAA supplementation on sarcopenia after stroke. We also found that nutritional support helps functional improvement during neurological recovery. These results suggest that comprehensive rehabilitation intervention combined with BCAA supplementation could be a helpful option during the critical period of post-stroke neurological recovery.

## Introduction

Stroke-related sarcopenia occurs due to a variety of factors, such as brain damage, systemic catabolic state, skeletal muscle imbalance, and malnutrition (1). Rosenberg defined “sarcopenia” as an age-related decline in lean body mass (LBM), muscle mass, and function (2); similar changes are also seen after stroke. Brain lesions in patients with a stroke lead to complex systemic metabolic changes, characterized by weight loss and anabolic catabolic imbalance (3). According to current studies, it was estimated that the incidence of sarcopenia in patients with stroke has been reported to range from 14 to 54% (4).

Stroke-related sarcopenia can be distinguished from sarcopenia itself (1), and it is related to direct catabolic signals from stroke injury, a rapid decline in muscle mass, and structural muscle alterations. However, multiple factors related to stroke accelerate functional disabilities and decrease muscle mass that causes stroke-related sarcopenia.

In stroke, brain lesions cause various neurological deficits and functional impairments. The US National Longitudinal Health and Retirement Study confirmed the findings of previous studies that stroke is the most frequent cause of functional impairment (5). Approximately 50% of patients are disabled, and 30% of the remaining patients are unable to walk (1, 6). Swallowing difficulties are present in 30–50% of patients after stroke, leading to malnutrition (4, 7, 8).

Brain lesions also activate the systemic catabolic pathway. Muscle structural changes develop within hours after stroke, followed by a rapid reduction in muscle mass. As early as 3 weeks after stroke, a significant decrease in muscle mass can be observed (9). Within 1 year after stroke, up to 3% of the lean mass of the paretic limb is lost (10) and within 6–12 months after stroke, a decrease in the muscle volume of the paretic limb of up to 24% has been observed (11).

Furthermore, stroke-related sarcopenia may accelerate these disadvantages and the massive burden in all areas, such as delaying the poststroke rehabilitation period, worsening the patients' functional recovery (3), lengthening the hospital stay, and increasing economic burden (4, 12). Thus, strategies to prevent the development of sarcopenia after stroke are important.

To improve the functions of patients with stroke, various rehabilitation interventions have been implemented (13, 14). Previously recommended, management of stroke-related sarcopenia should include rehabilitation intervention and nutritional support. Recent review studies have emphasized the consumption of branched-chain amino acids (BCAAs) by sarcopenic patients (15). BCAAs are essential amino acids that induce a muscle protein anabolic response and stimulate muscle mass growth (16). It could also resolve malnutrition after stroke. However, few studies have investigated the BCAA supplementation and stroke-related sarcopenia.

As neurological recovery after stroke does not follow a linear but a logarithm pattern (13), early appropriate, multiple rehabilitation interventions are important for maximum functional improvement after stroke (14). We hypothesized that consuming BCAAs after stroke could prevent rapid muscle loss after stroke and lead to functional improvement during the important neurological recovery periods.

The aim of this study was to investigate the effect of BCAA supplementation on sarcopenia after stroke. We also evaluated the effect of BCAA on functional improvement during the intensive rehabilitation period after stroke.

## Materials and Methods

### Participants

Patients with subacute stroke who had stroke-related disabilities and needed intensive rehabilitation intervention were registered. The following patients were included:

(1) Those with subacute stroke within 3 months after stroke.
(2) Those with stroke-related disabilities (> 3 mRS, which correlate moderate disability).
(3) Those with neurologically stable condition, without reattack and reinfarction-related stroke.
(4) Those who were medically stable and able to receive intensive rehabilitation therapy (>3 h/day).

The following patients were excluded:

(1) Those with chronic kidney disease (> CKD stage 2, which represent kidney damage with mild loss of kidney function).
(2) Those with elevated blood urea nitrogen (BUN) levels (over the normal range: 6–20 mg/dl).
(3) Those with elevated creatine (Cr) levels (over the normal range: 0.5–1.2 mg/dl).
(4) Those with uncontrolled infectious status, which needs antibiotic therapy and unstable vital sign (high temperature over 37.8°C, and lower or elevated heart rate, and pulse).

The study group was prospectively enrolled for ingesting BCAAs between January 2021 and June 2021; since the BCAAs were supplied through the nutrition support team during feeding time, this study could not be blinded with respect to the administration of the BCAAs. Patients whose age and stroke lesions were similar to those in the study group were enrolled in the control group through a retrospective medical record review between 2019 and 2020; those for whom dual-energy X-ray absorptiometry (DEXA) had not been performed were also excluded. This is a non-randomized, non-blinded as well as age, sex, and stroke lesion-matched comparative study. All patients were selected from the department of a rehabilitation unit in a single hospital and treated with the same intensive routine rehabilitation therapy for stroke. The study protocol was approved by the institutional review board of our hospital (DC21RISI0014).

### Interventions

Patients in the study group were given 30 g of Seniup^®^ (Enterogenomics Co., Daejeon, Korea) dietary supplement powder containing 6 g of BCAAs (2,976 mg of l-leucine, 1,512 mg of l-isoleucine, and 1,512 mg of l-valine) to be taken twice a day for 1 month. These BCAAs were provided by the nutrition team during feeding time. If the patient was fed through an enteral tube due to swallowing difficulty, the BCAAs were administered *via* the l-tube after being mixed with water. If the patient was prescribed a therapeutic diet, such as a thickener with water, the BCAAs were administered by mixing them with water and the thickener. The patients' target calories were calculated regularly by the nutritional support team according to the patients' condition and feeding materials in our institution. Because patients were received intensive rehabilitation therapy, their required energy was calculated using the Harris-Benedict equation (HBE), which was equal to 110% of the estimated amount of basal energy expenditure (BEE). Thus, each patient was provided almost 30 kcal/kg per day.

All patients in the study group and the control group were treated with a personalized, routine intensive inpatient poststroke rehabilitation therapy (17, 18), which included physiotherapy, occupational therapy, swallowing therapy, speech therapy, and modality. The patients also received regular functional evaluations every month during the intensive rehabilitation treatment period.

### Evaluations

#### Sarcopenia Evaluation

The sarcopenia status was assessed using handgrip strength and DEXA. Handgrip strength is a reliable and simple marker of muscle strength of the upper extremities (19). The highest test reliability for the unaffected side handgrip test was obtained when the mean of 3 measurements was used (20). DEXA is a standard tool for measuring muscle mass and is the most frequently used instrument for the assessment of body composition in patients with stroke (21). The skeletal muscle index (SMI) was defined as appendicular lean mass as assessed by DEXA divided by height in meters squared.

Unfortunately, the cutoff values for stroke-related sarcopenia have not yet been established; thus, we used the values of the definition of sarcopenia for Asian patients according to the Asian Working Group for Sarcopenia (15). Low muscle strength was defined as a handgrip strength <28 kg for men and <18 kg for women. The criteria for poor physical performance were a 6-m walk speed of <1.0 m/s and a height-adjusted muscle mass of <7.0 kg/m^2^ in men and <5.4 kg/m^2^ in women according to DEXA. Gait speed is an important diagnostic criterion for sarcopenia; however, the applicability of the assessment of gait speed is limited in patients with stroke. The short physical performance battery t(SPPBT) is another well-established scale for assessing physical performance and functions in sarcopenia (22). However, the SPPBT could not be administered in this study because most of the enrolled patients could not stand independently. Therefore, functional status was evaluated using various functional evaluation tools developed and verified for patients with stroke instead of the gait speed or SPPBT.

#### Functional Evaluation

We evaluated functional status related to stroke using the Korean version of modified Barthel index (K-MBI), Berg balance scale (BBS), functional ambulatory category (FAC), and manual function test (MFT), which have been well-established for patients with stroke. The K-MBI is used to assess performance in 10 basic activities related to self-care and mobility, with a score ranging from 0 to 100 and lower scores indicating greater dependency (23). The BBS is used to evaluate a patient's ability to safely balance during a series of predetermined tasks. The score ranges from 0 to 56; a score of 56 indicates functional balance and a score below 45 indicates that the individual may be at greater risk of falling (24). The FAC is a functional walking test that evaluates ambulation ability. This 6-point scale determines ambulation status by determining how much energy the patient requires when walking, regardless of whether or not they use an assistive device (25). The MFT measures gross and fine motor dexterities in the upper extremities on a scale of 0 to 32, and its reliability is considered excellent (26).

Swallowing function was evaluated using the functional dysphagia scale (FDS) and penetration aspiration scale (PAS) (27) based on the results of the video fluoroscopic swallowing study (VFSS). The FDS (28) was developed to quantify the severity of dysphagia; it correlates well with the American Speech-Language-Hearing Association national outcome measurement system criteria. The higher the score, the more severe the dysphagia. The PAS evaluates airway invasions and has a maximum score of 8 points. Scores are determined based on the depth to which material passes into the airway and based on whether material passes below the vocal fold and any effort to make eject the material. The penetration category corresponds to level 3 to 5 on the scale, and levels 6 to 8 According to the results of the VFSS, the patients' feeding methods were decided as follows: non-oral feeding, limited diet, or normal regular diet. Feeding status was presented as a dysphagia outcome and severity scale (DOSS) (29). It consists of 7-point levels (1–7), with higher scores indicating a normal diet. Level 1 indicates severe dysphagia that patients could not be fed orally.

All evaluations, including sarcopenic and functional status, were part of routine evaluations in the rehabilitation unit, thus, they were measured in both the study and control groups every month. Patients in the study group were evaluated before and after 4 weeks of BCAA treatment. All assessments were done by blinded therapists who were unaware of the study protocol.

### Statistical Analysis

SPSS 24.0 (IBM Co., Armonk, NY, USA) for Windows was used for statistical analysis. The Student's *t*-test and the chi-square test were used to compare the study and control groups. The paired *t*-test was used to compare the treatment effects measured before and 1 month after therapy in each group. Correlation analysis between sarcopenia and functional status was assessed using Pearson's correlation coefficients. Univariate and multivariate regression analyses were performed to determine which factors affected poststroke sarcopenia. A *p* < 0.05 was considered statistically significant. Since patients in the control group were enrolled *via* a retrospective chart review, the sample size could not be calculated before the study. Therefore, we have analyzed the power calculation for changes in the SMI and functional status between the two groups, and it showed a high effective sample size (>80%).

## Results

All enrolled patients aged above 65 years and had experienced a subacute stroke phase, within 3 months of the stroke onset. In total, 54 patients were enrolled, with 27 patients from each of the two groups. All patients were recruited from a single rehabilitation unit and received the same rehabilitation therapy except BCAA supplementation. Table 1 shows the demographic characteristics of the patients in both groups. There was no significant difference between the two groups in the initial evaluations and demographic factors. According to the 2019 sarcopenia criteria, most of the enrolled patients already showed decreased SMI on DEXA and decreased handgrip strength in the baseline evaluation (15).

**Table 1:** Demographic characteristics of patients in both groups.

**Characteristic**	**Study group (*n* = 27)**	**Control group (*n* = 27)**	***P*-value**
Age (years)	76.52 ± 7.71	75.93 ± 8.53	0.790
Gender (male/female)	14/13	14/13	0.409
NIHSS (score)	13.7 ± 9.1	15.1 ± 7.3	0.285
Diabetes (n, %)	13 (48.15)	15 (55.56)	0.514
Hypertension (n, %)	16 (59.26)	17(62.96)	0.306
Stroke type Ischemic/hemorrhagic	15/12	15/12	0.903
Stroke lesion Cortical/subcortical/brainstem	11/8/8	10/9/8	0.534
Stroke side Right/left	15/12	14/13	0.627
**Initial evaluations**			
Days from stroke onset (Initial evaluation, days)	48.10 ± 21.68	51.34 ± 23.71	0.679
Cognition–MMSE	7.29 ± 6.91	9.05 ± 7.41	0.627
**Swallowing function**			
Non-oral feeding (n, %)	10 (37.04)	8 (29.63)	0.094
Limited diet (n, %)	13 (48.15)	16 (59.26)	0.328
Regular diet (n, %)	4 (14.81)	3 (11.11)	0.771
Albumin	3.54 ± 0.41	3.41 ± 0.42	0.258
BMI (kg/m^2^)	19.51 ± 4.24	17.72 ±5.53	0.467
BMD	−2.71 ± 1.09	−2.38 ± 1.28	0.309
Handgrip strength (kg)	12.13 ±5.72	13.40 ± 12.43	0.174
**DEXA**			
SMI (kg/m^2^)	4.70 ± 0.66	4.73 ± 0.70	0.902
Est. VAT area	107.9 ± 46.76	103.59 ± 34.98	0.703
LBM Affected upper extremity (g)	1,511.08 ±3 25.73	1,609.74 ± 335.84	0.278
Intact upper extremity (g)	1,595.53 ± 328.51	1,711.04 ± 330.64	0.204
Affected lower extremity (g)	4,432.34 ± 860.63	4,724.41 ± 975.46	0.249
Intact lower extremity (g)	4,494.71 ± 880.02	4,913.93 ± 983.88	0.105

*Values are mean ± SD, or numbers. BMI, body mass index; BMD, bone mineral density; DEXA, dual energy X-ray absorptiometry; SMI, skeletal muscle index; LBM, lean body mass*.

**Figure 1:**
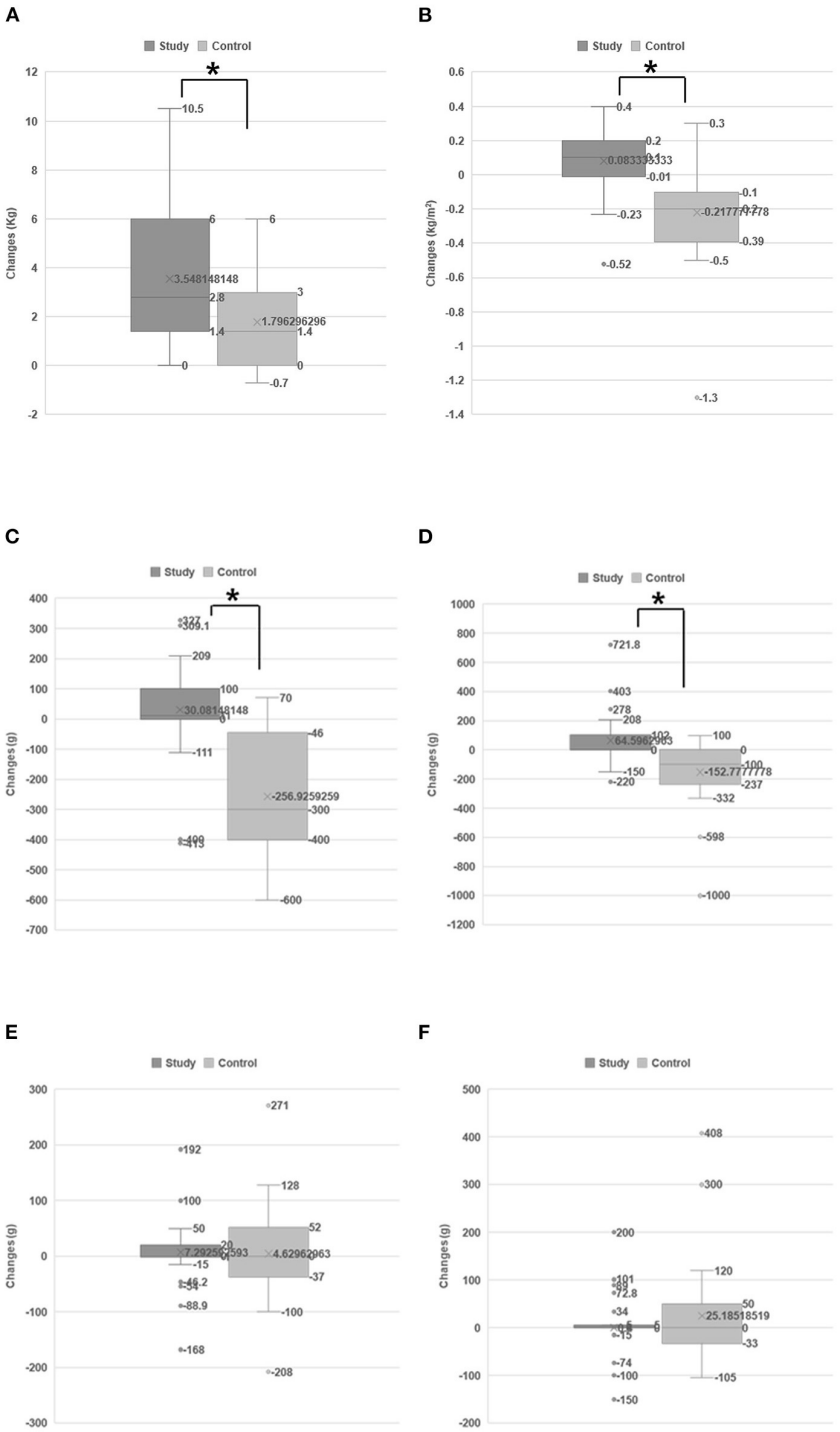
Changes in sarcopenic status between two groups. The changes of handgrip strength **(A)** and SMI **(B)**. The study group improved significantly in terms of handgrip strength **(A)** and SMI **(B)** than the control group. The changes of LBM on each extremity: affected lower extremity **(C)**, intact lower extremity **(D)**, affected upper extremity **(E)**, and intact upper extremity **(F)**. The LBM of the affected lower extremities of the control group was markedly, significantly decreased after treatment **(C)**, and the intact lower extremities also showed a decreased LBM **(D)**. However, rather than decreasing, the LBM of the affected lower extremities of the study group patients was slightly, significantly increased **(C)**. *p < 0.05 according to independent *t*-test.

**Figure 2:**
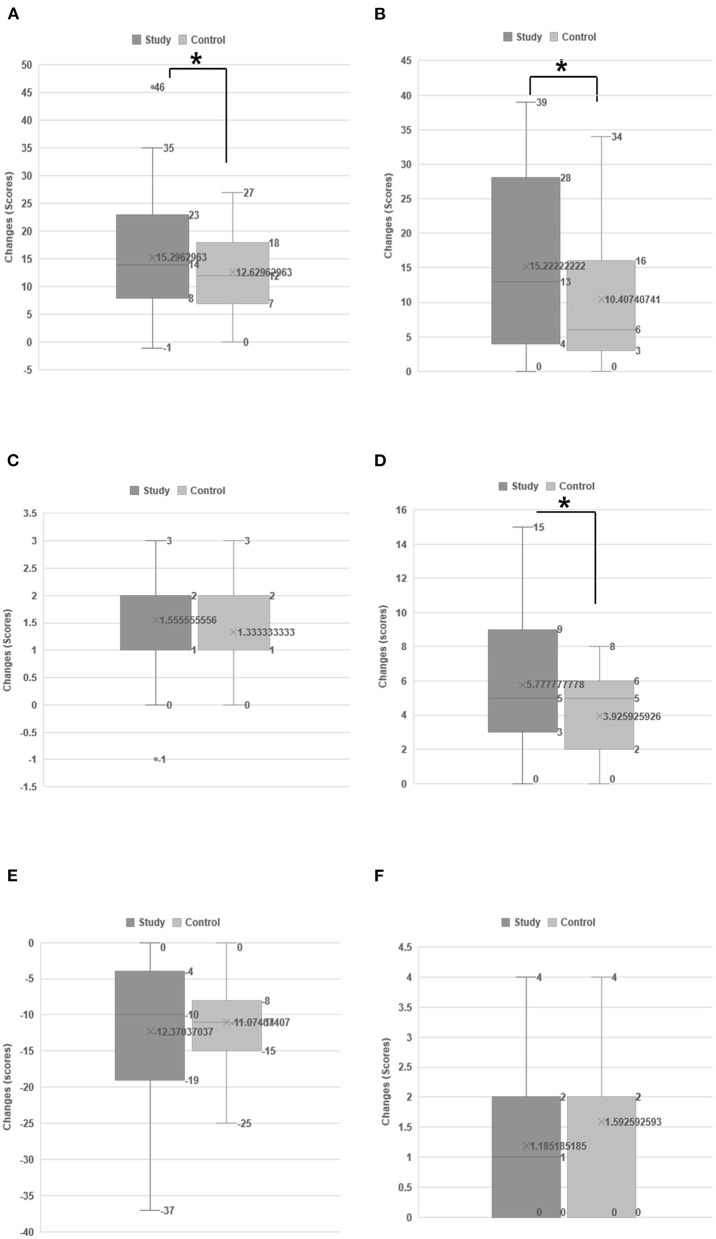
Changes in the functional status between two groups. Changes in the functional status between two groups: Activities of daily living using MBI **(A)**, the Berg balance scale **(B)**, functional ambulation category **(C)**, upper extremity function using the manual function test **(D)**, swallowing function using the functional dysphagia scale **(E)**, and dysphagia outcome and severity scale **(F)**. Both groups showed functional improvement over time; however, the improvement in the study group was significantly greater than that in the control group. *p < 0.05 according to independent *t*-test.

Table 2 shows sarcopenia and functional evaluations before and after treatment in each group. The study group showed a slight increase in the handgrip strength and SMI after the intervention, but it was statistically significant. However, in the control group, SMI was slightly decreased. Both groups showed significant improvement in functional evaluations. Figures 1, 2 compared the changes in sarcopenia and functional evaluations between the two groups. After BCAA supplementation and intensive rehabilitation therapy, the study group showed significantly greater SMI than the control group, which showed significantly decreased SMI after therapy (Table 2 and Figure 1A).

**Table 2:** Sarcopenia and functional evaluations before and after treatment within the group.

**Parameters**	**Study group**			**Control group**		
**Sarcopenic evaluations**	**Pre-Tx**	**Post-Tx**	***P*-value**	**Pre-Tx**	**Post-Tx**	***P*-value**
Handgrip strength (kg)	12.13 ± 5.72	15.68 ± 6.22^*^	0.021	13.40 ± 12.43	15.19 ± 7.08	0.085
**DEXA**
SMI (kg/m^2^)	4.68 ± 0.66	4.79 ± 0.69^*^	0.023	4.73 ± 0.70	4.51 ± 0.74^*^	0.039
Est. VAT area	107.9 ± 46.76	108.41 ± 42.45	0.602	103.59 ± 34.98	104.85 ± 37.09	0.451
**LBM**
Affected upper extremity (g)	1,511.08 ± 325.73	1,518.37 ± 138.50^*^	0.037	1,609.74 ± 335.84	1,604.37 ± 362.78	0.901
Intact upper extremity (g)	1,595.53 ± 328.51	1,602.93 ± 308.72^*^	0.040	1,711.04 ± 330.64	1,736.22 ± 341.48	0.128
Affected lower extremity (g)	4,432.34 ± 860.63	4,462.43 ± 904.04^*^	0.001	4,724.41 ± 975.46	4,467.48 ± 409.12^*^	<0.001
Intact lower extremity (g)	4,494.71 ± 880.02	4,559.30 ± 930.14	0.257	4,913.93 ± 983.88	4,607.52 ± 571.61^*^	0.004
**Functional evaluations**	**Pre-Tx**	**Post-Tx**	***P*** **-value**	**Pre-Tx**	**Post-Tx**	***P*** **-value**
Activities of daily living: K-MBI	27.19 ± 15.40	42.48 ± 20.42^*^	<0.001	24.07 ± 13.61	37.33 ±17.52^*^	<0.001
Balance and gait - BBS	9.45 ± 12.74	24.67 ± 16.67^*^	<0.001	6.48 ± 5.94	16.89 ± 15.26^*^	<0.001
Gait function- FAC	1.70 ± 0.91	3.26 ± 1.31^*^	0.021	1.48 ± 0.70	2.81 ± 1.30^*^	0.030
Upper ext. function - MFT	15.07 ± 7.21	20.85 ±6.31^*^	<0.001	15.22 ± 7.78	19.15 ± 7.57^*^	0.041
Swallowing function -FDS	35.63 ± 15.30	23.26 ± 11.47^*^	<0.001	39.85 ± 12.43	28.78 ± 14.35^*^	<0.001
Swallowing function - PAS	5.67 ± 2.45	3.89 ± 2.40^*^	0.034	6.44 ±1.78	4.89 ± 1.97^*^	0.048
Feeding materials - DOSS	4.04 ± 1.74	5.22 ± 1.48^*^	<0.001	3.04 ±1.65	4.63 ± 1.47^*^	<0.001

*Values are mean ± SD. DEXA, dual-energy X-ray absorptiometry; SMI, skeletal muscle index; LBM, lean body mass; K-MBI, Korean version of modified Barthel index; BBS, Berg balance scale; FAC, functional ambulatory category; MFT, manual function test; FDS, functional dysphagia scale; PAS, penetration aspiration scale; DOSS, dysphagia outcome and severity scale*,

* *p < 0.05 according to paired t-test*.

The DEXA system obtained the LBM from each of the four extremities (affected [hemiparetic side, weak] upper and lower extremities and intact upper and lower extremities). Among these, the affected (hemiparetic side) lower extremities in patients in the control group demonstrated markedly, significantly decreased LBM after conventional rehabilitation therapy. The intact lower extremities also showed significantly decreased LBM. In contrast, the LBM of the affected lower extremities of the study group patients increased slightly but significantly (Table 2 and Figure 1B).

Both groups exhibited functional improvement over time, but the improvement in the study group was significantly greater than that in the control group (Figure 2). Based on the FAC, BBS, and K-MBI scores, 46% of patients in the study group and 37% of those in the control group were unable to walk due to stroke at the initial evaluation and were able to walk again with assistance after treatment (Table 2). Swallowing function also improved over time according to the DOSS, FDS, and PAS. After treatment, 59% of patients in the study group and 53% of those in the control group who were initially fed *via* Levin tube could feed orally (Table 2); however, this difference was not statistically significant between the two groups (Figure 2).

Table 3 shows a pairwise correlation analysis between sarcopenia and functional status. It shows a significant relationship between changes in the SMI and improvements in functional scores, especially ambulatory function, in the study group (BBS; Pearson's correlation coefficient: 0.461, *p* = 0.016, FAC; Pearson's correlation coefficient: 0.543, *p* = 0.003). The initial SMI was not related to functional changes in either group.

**Table 3:** Pairwise correlations between function and sarcopenia status.

**Two test**	**Total**		**Study group**		**Control group**	
	**Pearson's correlation coefficient**	***P*-value**	**Pearson's correlation coefficient**	***P*-value**	**Pearson's correlation coefficient**	***P*-value**
Initial SMI - Δ K-MBI	0.1986	0.150	0.279	0.159	0.104	0.604
Initial SMI- Δ BBS	0.0299	0.830	0.100	0.617	0.04	0.412
Initial SMI- Δ FAC	0.1974	0.153	0.235	0.238	0.165	0.835
Initial SMI- Δ MFT	0.1624	0.241	0.290	0.143	0.013	0.949
Initial SMI- Δ Grip strength	0.181	0.191	0.348	0.074	−0.023	0.908
Initial SMI- Δ FDS	0.0525	0.707	0.194	0.334	−0.178	0.374
Initial SMI- Δ PAS	0.0561	0.687	−0.031	0.879	0.141	0.483
Initial SMI - Δ DOSS	0.0124	0.902	0.228	0.264	−0.175	0.383
ΔSMI - Δ K-MBI	0.293^*^	0.032	0.265	0.182	0.296	0.134
Δ SMI- Δ BBS	0.296^*^	0.030	0.461^*^	0.016	0.049	0.963
Δ SMI- Δ FAC	0.305^*^	0.025	0.543^*^	0.003	0.010	0.807
Δ SMI- Δ MFT	0.267	0.051	0.276	0.163	0.053	0.791
Δ SMI- Δ Grip strength	0.278^*^	0.042	0.445^*^	0.020	−0.118	0.559
Δ SMI- Δ FDS	−0.160	0.250	−0.137	0.496	−0.166	0.409
Δ SMI- Δ PAS	−0.130	0.347	−0.204	0.307	0.022	0.915
ΔSMI - Δ DOSS	−0.150	0.282	−0.093	0.641	−0.019	0.925

*Values are mean ± SD. K-MBI, Korean version of the modified Barthel index; BBS, Berg balance scale; FAC, functional ambulatory category; MFT, manual function test; FDS, functional dysphagia scale; PAS, penetration aspiration scale; DOSS, dysphagia outcome and severity scale; SMI, skeletal muscle index*,

* *p < 0.05 by Pearson's correlation coefficient*.

Univariate regression analysis (Table 4) revealed that patients in the study group with higher initial functional scores, higher grip strengths, and higher improvements in functional scores had significantly higher SMI after receiving both intensive rehabilitation therapy and BCAA supplementation. Among all enrolled patients between the study and control groups, an initial higher functional score and better functional improvement were also significantly related to SMI changes. Patients in the control group did not show significant results in univariate analysis.

**Table 4:** Univariate linear regression analysis related factors for changes in SMI (dependent variable: Δ SMI).

**Variables**	**Estimated**	**Total**				**Estimated**	**Study group**			
		**95% CI**		**R^**2**^**	***P*-value**		**95% CI**		**R^**2**^**	***P*-value**
		**Lower**	**Upper**				**Lower**	**Upper**		
initial K-MBI	0.0056	0.003	0.010	0.080^*^	0.039	0.0110	0.004	0.018	0.274^*^	0.005
Initial BBS	0.0117	0.005	0.019	0.171^*^	0.002	0.0238	0.007	0.041	0.246^*^	0.009
Initial FAC	0.1233	0.031	0.216	0.122^*^	0.01	0.1994	0.053	0.346	0.240^*^	0.010
Initial MFT	0.0059	−0.005	0.017	0.023	0.274	0.0161	0.003	0.030	0.193^*^	0.022
Initial DOSS	0.0589	0.016	0.101	0.129^*^	0.008	0.0820	0.019	0.145	0.23^*^	0.012
Initial SMI	−0.0167	−0.136	0.102	0.002	0.779	−0.0172	−0.186	0.152	0.002	0.836
Initial grip strength	0.0083	−0.005	0.021	0.031	0.203	0.0174	0.008	0.034	0.158^*^	0.040
Δ K-MBI	0.0089	0.001	0.017	0.086^*^	0.032	0.0106	−0.005	0.026	0.070	0.182
Δ BBS	0.0080	0.001	0.015	0.088^*^	0.030	0.0133	0.003	0.024	0.213^*^	0.016
Δ FAC	0.0949	0.013	0.177	0.093^*^	0.025	0.1861	0.068	0.305	0.295^*^	0.003
Δ MFT	0.0230	−0.001	0.046	0.071	0.051	0.0326	−0.014	0.079	0.076	0.163
Δ FDS	−0.0057	−0.015	0.004	0.026	0.248	−0.0068	−0.027	0.014	0.019	0.496
Δ DOSS	−0.0376	−0.107	0.032	0.022	0.282	−0.0226	−0.121	0.076	0.009	0.641
Δ Grip strength	0.0301	0.001	0.059	0.077^*^	0.042	0.0694	0.012	0.127	0.198^*^	0.020

*Values are mean ± SD. K-MBI, Korean version of modified Barthel index; BBS, Berg balance scale; FAC, functional ambulatory category; MFT, manual function test; FDS, functional dysphagia scale; DOSS, dysphagia outcome and severity scale; SMI, skeletal muscle index*;

* *p < 0.05 according to univariate linear regression analysis*.

Multivariate regression analysis (Table 5) was used for variables with a *p* < 0.05 in the univariate analysis, and the variables were entered into the model selection procedure using a backward stepwise process. According to the multivariate analysis, a high initial MBI score and increased handgrip strength were independent factors that predicted an increase in the SMI score in the study group. Among all enrolled patients, the initial BBS score and changes in the FAC score were independent factors predicting an increase in the SMI (Table 5).

**Table 5:** Multivariate linear regression analysis related factors for changes in SMI.

**Outcome/independent predictors**	**95% CI**		***P*-value**	**Adjusted R^**2**^**
	**lower**	**Upper**		
**Δ** **SMI (Total patients)**				
Initial BBS	0.004	0.018	0.002	0.245
Δ FAC	0.008	0.161	0.031	
**Δ** **SMI (Study group)**				
Initial K-MBI	0.002	0.017	0.01	0.396
Δ Grip strength	0.003	0.107	0.038	

*Values are mean ± SD. SMI, skeletal muscle index; BBS, Berg balance scale; FAC, functional ambulatory category; K-MBI, Korean version of the modified Barthel index*.

There were few complications during BCAA supplementation and rehabilitation. A male patient who aged 65 years suffered from acute renal failure; his creatine level increased from 0.9 to 2.1, and the BUN level increased from 27 to 64. Another male patient who aged 79 years suffered from rhabdomyolysis; he also had several medical problems such as arterial fibrillation, diabetes, and anemia of chronic disease. The BCAA supplementation was stopped in these patients, and their conditions were restored. However, a direct relationship between these complications and BCAA ingestion could not be verified.

## Discussion

This study aimed to evaluate the effect of BCAA supplementation on stroke-related sarcopenia as well as the relationship between BCAA supplementation and functional recovery. Our results revealed that the study group improved significantly in terms of SMI and functional status after BCAA supplementation and intensive rehabilitation therapy than the control group. Recent stroke rehabilitation studies have suggested that stroke recovery does not follow a linear pattern; thus, maximal, multifactorial, comprehensive therapy should be used to treat patients with stroke during the critical recovery period (13, 14). We suggest that BCAA supplementation would be a helpful adjuvant therapy during the intensive stroke rehabilitation period for achieving good functional outcomes.

### Stroke-Related Sarcopenia: Possible Mechanism and Prevention Strategies

After stroke, the brain lesion subsequently interrupts the corticospinal and corticobulbar tracts, resulting in weakness of the contralateral extremities and swallowing difficulty. Impairment of physical activities is accompanied by structural changes in skeletal muscle, resulting in disuse atrophy. Brain lesions also cause systemic activation of catabolic pathways, which are responsible for the apoptotic and proteolytic reactions in the muscles (1). These complex, systemic metabolic changes and the oxidative stress produced after stroke suppress protein synthesis, resulting in poststroke sarcopenia and impairment in brain recovery (8, 30). Additionally, the energy requirements of patients increase after stroke during the intensive rehabilitation period. Undernutrition and physical inactivity due to weakness in patients with stroke have been significantly associated with sarcopenia and poor clinical outcomes. In contrast, nutritional support has been shown to strongly enhance the functional outcomes of patients with stroke by preserving muscle and fat masses (8, 31, 32). Furthermore, better physical functions after stroke could shorten hospitalization stays and decrease the patients' economic burdens (12).

“Rehabilitation nutrition,” presented by Wakabayashi (33), is a concept combining both rehabilitation and nutrition care management. This concept further improves outcomes in elderly patients with stroke with malnutrition and sarcopenia. A variety of nutritional supplements are available worldwide. Among them, BCAAs provide necessary amino acids that cannot be synthesized in the human body and are responsible for building muscle mass; thus, BCAA intake would be an essential ingredient for nutritional support. Furthermore, BCAAs could play an important role in providing anti-inflammatory and anabolic effects after stroke (32). Our results are consistent with previous results showing that protein supplementation is significantly associated with more favorable functional improvement and prevents decreased skeletal muscle mass in patients with stroke (32, 34). In addition, dysphagia after stroke causes malnutrition and is associated with a poor prognosis (8). The BCAA powder used in our study could be applied *via* a Levin tube if the patient could not feed orally. Thus, the early detection of sarcopenia after stroke and the combination of BCAA supplementation and intensive rehabilitation can promote the restoration of muscle mass and accelerate neurological recovery.

### Effect of BCAA Supplementation on Sarcopenic Status After Stroke

Compared with the control group, the SMI of the study group significantly increased after BCAA supplementation and intensive rehabilitation therapy. After stroke, brain lesions activate the systemic catabolic pathway, and muscle structural changes lead to a rapid reduction in muscle mass. Furthermore, physical inactivity due to weakness and malnutrition caused by dysphagia accelerate this muscle loss. Previous studies have also demonstrated that muscle loss occurs over time after stroke. Only 3 weeks after stroke, a significant decrease in muscle mass was reported (10) and the lean mass of the paretic limb has been shown to decrease up to 24% within 6–12 months of stroke onset (11). Recently, it has been reported that up to 50% of older poststroke patients are diagnosed with sarcopenia as defined by the Asian Working Group for Sarcopenia (15, 32). Another study revealed that the ipsilateral leg, which was not affected by the brain lesion, also loses muscle mass (9). Our results are consistent with these findings; we enrolled patients within 3 months after stroke, and 89% of them already showed a decreased SMI based on initial evaluations. In addition, 32% of the study group and 60% of the control group had aggravated sarcopenia (decreased SMI scores) after stroke despite undergoing rehabilitation treatment.

In contrast to previous studies, we evaluated SMI and LBM for each of the four extremities (affected and intact upper and lower extremities). Among these, the affected lower extremities of the patients in the control group demonstrated a significantly decreased LBM despite undergoing rehabilitation treatment. The LBM decrease was more prominent in the weight-bearing affected lower extremity muscles than in the non-weight-bearing upper extremity muscles. In contrast, the LBM of the affected lower extremities of the study group patients significantly increased after the intervention. To our knowledge, this study is the first to show the effect of BCAAs on preventing a decrease in muscle mass in the affected lower extremities, particularly by analyzing each of the extremities individually. Thus, proactive management for preventing muscle mass loss, such as BCAA supplementation, neuromuscular electrical stimulation, or other intensive therapy, should be conducted after stroke.

### BCAA Supplementation and Functional Improvement

We provided 12 g of BCAAs per day for 1 month, which is similar to the amount used in previous studies (35, 36). Previous studies have demonstrated the pharmacological effects of BCAAs, such as anti-inflammatory effects, improved liver cirrhosis, and reduced incidence of heart failure with hypoalbuminemia (32, 35, 37). This study was conducted on patients with stroke who were admitted to rehabilitation centers; thus, if the patients had abnormal laboratory findings, such as elevated C-reactive protein, white blood cell count, or liver enzymes, we managed them properly. Thus, we could not evaluate the effect of BCAAs on inflammation or liver enzymes. However, our results revealed that after controlling the inflammation and laboratory abnormalities, BCAA supplementation could have a positive effect on functional status in patients with stroke-related sarcopenia.

In this study, we divided patients' functions into various domains, such as ambulation, activities of daily living, and swallowing function. As mentioned earlier, both groups improved functionally over time; when compared with the control group, the study group improved significantly in ambulation and activities of daily living. Correlation analysis showed a significant correlation between improvement of functional status and increased SMI. For patients in the study group with higher initial functional scores, the univariate regression analysis revealed a positive effect on sarcopenia after stroke was achieved with BCAA supplementation. Our results support the potential benefit of BCAA supplementation in producing functional improvement through increasing SMI.

### Study Limitations

This study has several limitations. We were unable to perform a randomized control trial since the BCAAs were administered during feeding time, and we did not use placebo drugs; thus, this study could not be blinded in regard to the use of BCAAs. Instead, we enrolled a control group whose age and stroke-lesions were similar to those of the study group *via* meticulous medical chart review, achieving a high effective sample size. All patients were selected from a single rehabilitation unit and received the same rehabilitation therapy. In addition, most (~89%) of enrolled patients were sarcopenic, but we could not determine whether these patients had sarcopenia before stroke onset or whether it had developed after stroke. Thus, we could not evaluate the effect of sarcopenia on the stroke attack. Our intervention period was only 1 month; thus, the SMI scores did not dramatically change before and after treatment evaluation; however, we observed greater functional status changes than with the SMI. A randomized study with a longer intervention period is needed to further validate these results.

## Conclusion

Our results showed a positive effect of BCAAs supplementation on stroke-related sarcopenia. The patients in the study group improved significantly in terms of SMI and functional status after BCAAs supplementation and intensive rehabilitation therapy than the control group. Stroke leads to complex systemic metabolic changes; thus, loss of muscle mass and malnutrition frequently occur after stroke. Besides, energy requirements are increased in patients with stroke during intensive rehabilitation period and malnutrition could affect neurological recovery. Thus, proper strategies involving rehabilitation interventions and nutritional support are important for maximum functional improvement after stroke. We suggest that BCAAs supplementation would be a helpful, adjuvant therapy at a critical period of poststroke neurological recovery during intensive stroke rehabilitation intervention.

## Data Availability Statement

Individualized data cannot be released due to personal data protection however the datasets generated for this study are available on request to the corresponding author.

## Ethics Statement

The studies involving human participants were reviewed and approved by Daejeon St. Mary's Hospital, the Catholic University of Korea. The patients/participants provided their written informed consent to participate in this study.

## Author Contributions

MP and SoL contributed to the design and conception of the work and wrote the manuscript. EC and SaL performed the experiments and contributed materials. MP and JL analyzed the data. All authors contributed to the article and approved the submitted version.

## Funding

This study was supported by the Catholic Medical Center Research Foundation made in the program year of 2019.

## Conflict of Interest

The authors declare that the research was conducted in the absence of any commercial or financial relationships that could be construed as a potential conflict of interest.

## Publisher's Note

All claims expressed in this article are solely those of the authors and do not necessarily represent those of their affiliated organizations, or those of the publisher, the editors and the reviewers. Any product that may be evaluated in this article, or claim that may be made by its manufacturer, is not guaranteed or endorsed by the publisher.
